# The Uncharted Territory of the New Obesity Drugs in Users Without Obesity: A Sociomedical Perspective

**DOI:** 10.1002/oby.70069

**Published:** 2025-11-12

**Authors:** Fernanda Baeza Scagliusi, Bruno Gualano, Pernille Andreassen, Cindi SturtzSreetharan, Sissel Due Jensen, Alexandra Brewis

**Affiliations:** ^1^ Department of Nutrition, Research Group in Eating, Corporalities and Culture (GPAC) University of São Paulo School of Public Health São Paulo São Paulo Brazil; ^2^ Department of Clinical Medicine, Center for Lifestyle Medicine (CMEV) University of São Paulo School of Medicine São Paulo São Paulo Brazil; ^3^ The Danish National Center for Obesity, Aarhus University Hospital Aarhus Jutland Denmark; ^4^ Arizona State University School of Human Evolution and Social Change, Center for Global Health Tempe Arizona USA; ^5^ Department of Public Health Aarhus University Aarhus Jutland Denmark; ^6^ Steno Diabetes Center Aarhus, Aarhus University Hospital Aarhus Jutland Denmark

**Keywords:** anti‐obesity drugs, obesity, off‐label use, slimness, sociomedical aspects

## Abstract

The off‐label use of glucagon‐like peptide‐1 receptor agonists (GLP‐1ra) among people without obesity and/or diabetes is rapidly expanding, despite a lack of clinical justification or safety data in this population. This Perspective explores the sociomedical dimensions of this trend, highlighting key research gaps and emerging hypotheses around the off‐label use of GLP‐1ra to an individual goal of slimness rather than health. We propose that off‐label GLP‐1ra use by people with normative or mildly elevated weight reveals deeper tensions between scientific progress, stigma, and cultural ideals of the body. Understanding this phenomenon requires moving beyond biomedical efficacy to interrogate the psychological, behavioral, and sociopolitical implications of pharmacological thinness. Cross‐cultural and intersectional approaches from the social sciences are key to understanding this emergent phenomenon.

## 1 Introduction

Glucagon‐like peptide‐1 receptor agonists (GLP‐1ra) have ushered in a new era in the treatment of obesity, offering unprecedented levels of weight loss with relatively manageable side‐effect profiles for individuals with obesity and/or type 2 diabetes [1, 2]. These drugs have been widely heralded by clinicians, lay people, and media as game‐changing therapies.

However, there is a growing interest in their use by those who are neither diagnosed with obesity nor associated with metabolic disorders [3]. Rather, these drugs are used off‐label to become and stay slim. This complex set of decisions and behaviors by consumers creates a “moral economy” of the body, meaning that certain types of slim bodies can act to reduce negative judgments by others, secure social legitimacy, elevate social status, and otherwise achieve an aspirational self‐image [4, 5]. That is, slimness rather than health appears to be a goal, providing users with a pathway to significant social and emotional benefits. In a sociological framing, the body is not only shaped by drugs, but also made socially visible and legible through them.

This Perspective explores the uncharted sociomedical territory of this off‐label use of the new obesity drugs. We outline major domains of “unknowns” that demand interdisciplinary inquiry: clinical, psychological, behavioral, cultural, and regulatory. We also explain why cross‐cultural and intersectional research is crucial to the explanation of and predictions around these emerging patterns of off‐label use.

## 2 From Metabolic Treatment to Body Optimization

Originally developed to treat metabolic conditions such as obesity and type 2 diabetes [1, 2], GLP‐1ra have now entered the realm of lifestyle management. Through media amplification, celebrity endorsements, peer‐to‐peer promotion, and online sales, these drugs have acquired symbolic power well beyond the clinic. They are marketed as tools of self‐optimization, bodily control, and social worth. Additionally, the inherent shortcomings of body mass Index in diagnosing obesity [6] may broaden the target population for these drugs.

As Brewis and Trainer postulate [7], even extreme weight loss is no longer merely a biomedical outcome; it is a visible, performative achievement. In this moral economy, losing weight signals commitment, discipline, and conformity to dominant body and health norms. Conversely, failing to lose weight (or choosing not to try) is framed as a failure of responsibility. In this light, we ask if off‐label use could be seen as an “abuse” or a “rational” adaptation to pervasive body surveillance and weight stigma.

For many users without obesity, GLP‐1ra may offer the promise of bodily conformity without the burden of traditional self‐discipline. They may help navigate a landscape in which thinness confers professional and social advantage and where weight, even modestly above average, is pathologized and penalized [8, 9]. In this environment, the line between therapeutic intervention and aesthetic enhancement becomes increasingly blurred.

## 3 Mapping the Gaps

Despite the rapid rise of off‐label GLP‐1ra use, empirical data remain sparse. Key questions that are not only clinically imperative but also culturally and ethically urgent remain unanswered (Table 1 details some of these):

**TABLE 1:** Key questions to guide studies concerning off‐label GLP‐1ra use.

Domains	Guiding questions
Food and appetite	How does appetite suppression alter users' emotional relationship with food? Do they feel liberated, alienated, or distressed by reduced hunger? Do they think about food more or less frequently? Do they feel a loss of control with specific foods or eat more to manage emotions?
Side effects	What emotional and physical side effects do off‐label users feel? How do they feel about these side effects? How do they manage them? Are side effects experienced as problems or markers of weight loss success?
Body image and self‐worth	How does GLP‐1ra use affect body image and self‐worth over time? Does being thinner lead to greater self‐acceptance or raise the bar for what is “enough?” Do they feel more or less satisfied with their bodies? Do they feel mentally better or worse? Is the off‐label GLP‐1ra use associated with other cosmetic procedures or the use of other human enhancement drugs?
Behavioral changes	How have eating practices changed during or after use? Have episodes of binge eating, food avoidance, or food‐related anxiety increased or decreased? Have they skipped meals or restricted food to lose weight? Do they feel guilty after eating? Do they wish they did not need to eat?
Psychological impact	Are there signs of anxiety, depression, or disordered eating that coincide with use? Do they feel nervous, anxious, or experience guilt related to eating or body shape?
Medicalization and access	How are users accessing the drugs, through medical channels or other means? What roles do cost and medical gatekeeping play? Who recommended the drug? Was it a self‐directed decision?
Emotional dependence and discontinuation	Do users fear regaining weight more than they fear side effects? How central is the drug to their self‐image and regulation? What has happened (or would happen) if they stopped using this drug? If they stopped taking it, what were the reasons? Do they feel trapped in a lifelong drug use to maintain weight loss?
Connections with health and healthism	Are concerns with health promotion, longevity, and disease prevention factors contributing to drug use? Do the GLP‐1ra users feel pressured to take responsibility for their health? Do users feel more in control of their health?
Digital and social influence	What is the role of social media and digital culture in facilitating or normalizing GLP‐1ra use? How did they hear about the drug? Was social media an influence?
Moral hierarchies of weight loss methods	Is there a moral hierarchy of weight loss methods? Do users view GLP‐1ra use as more or less legitimate than other methods? Have users felt judged or unsupported when hearing that these drugs are not meant for people like them?
Identity and meaning	How do users explain the choice of using these drugs? Do they see themselves as patients, consumers, rebels, or conformists? How do they feel about using this drug? What would they tell someone who is just starting to use it?

*Who are these users?* What are their demographic, psychological, and social profiles? Are they disproportionately women, as media suggest? How do social class, occupation, and racial/ethnic identities shape motivations and access?

*How are the drugs accessed?* Are users obtaining GLP‐1ra through prescribers, informal channels, or digital platforms? What role does medical gatekeeping play in this new consumer market?

*What are the experiential outcomes?* Do off‐label users navigate the side effects differently? How do GLP‐1ra affect users' relationships with food, their bodies, and their identities? What emotional costs, such as shame, anxiety, or dependence, accompany the desired physical transformation?

*How do users make sense of their actions?* Do they view GLP‐1ra use as medical, cosmetic, or moral? Do they feel empowered by their decision, or trapped, conflicted, and judged?

*What broader pressures are at play?* How do neoliberal ideals of self‐discipline, healthism, and aesthetic success fuel demand for these drugs, even among those without clinical need?

These questions demand an intersectional analysis of off‐label GLP‐1ra use, linking individual choices to the socioeconomic and cultural norms of body image.

## 4 Moving Toward a Multinational, Intersectional Research Agenda

Off‐label GLP‐1ra use is not simply a deviation from clinical norms; it is an emergent phenomenon that reflects global shifts in the cultural politics of the body. It reveals how the pursuit of thinness has been medicalized, moralized, and commodified in ways that demand rigorous, interdisciplinary scrutiny.

A comprehensive understanding of this trend requires coordinated, multinational research capable of capturing the heterogeneous motivations, normative frameworks, and structural vulnerabilities that shape GLP‐1ra use across sociocultural contexts. Our team includes researchers studying the sociomedical contexts of off‐label GLP‐1ra use in Brazil, the US, Denmark, and Japan. Through such initial cross‐national comparison, we are seeing both generalities and important distinctions in how these drugs are being deployed for slimness.

In Brazil, for example, such drugs primarily function as instruments for navigating racialized and class‐based beauty standards; in the US, as technologies of neoliberal self‐discipline; in Japan as public health surveillance and biocitizenship; and in Denmark, as extensions of a biomedical culture marked by high institutional trust and strong public health regulation. Comparative cultural analyses are therefore essential to elucidate the global complexity of this phenomenon, particularly given that experiences of “being fat” vary widely across different sociocultural contexts [10]. We aim to broaden this multinational initiative by adding researchers and fostering aligned collaborations.

Equally crucial is an intersectional lens to identify commonalities and distinctions within any given cultural setting. Body size always intersects with gender, race, age, class, and geography, shaping who uses these drugs, how they are judged, and what outcomes they experience. Without this lens, we risk reducing GLP‐1ra off‐label users to simplistic, stigmatizing categories (e.g., noncompliant, vain, cheater, misinformed), while ignoring the structural forces that make pharmaceutical thinness seem not only desirable but socially necessary.

Only by integrating biomedical, sociological, and cultural perspectives can we begin to understand what the new obesity drugs are really doing in the individual body as well as the social body, where the main revolution visibly occurs.

## Conflicts of Interest

The authors declare no conflicts of interest.

## Data Availability

Data sharing not applicable to this article as no datasets were generated or analyzed during the current study.
